# Effect of taxonomy and feeding guilds on waterbirds of the Southern Caspian Sea, Iran

**DOI:** 10.1371/journal.pone.0334915

**Published:** 2026-02-26

**Authors:** Mohammad Hosein Sinkakarimi, Mehdi Hassanpour

**Affiliations:** 1 Department of Environmental Sciences, Faculty of Marine and Environmental Sciences, University of Mazandaran, Babolsar, Mazandaran, Iran; 2 Department of Environment, Provincial Directorate of Environment Protection, Golestan, Iran; King Fahd University of Petroleum & Minerals, SAUDI ARABIA

## Abstract

This study measured the concentrations of arsenic (As), cadmium (Cd), chromium (Cr), iron (Fe), manganese (Mn), nickel (Ni), lead (Pb), and zinc (Zn) in the secondary flight feathers of waterbirds wintering along the southern Caspian Sea coast of Iran, in February 2013. Target species included *Anas acuta*, *Anas platyrhynchos*, *Anser anser*, *Aythya ferina*, *Ciconia ciconia*, *Cygnus olor*, *Hydroprogne caspia*, *Mareca penelope*, *Mergus merganser*, *Phalacrocorax carbo*, and *Tringa totanus*. Element concentrations ranged from highest in *P. carbo* (Pb 5.65, Cd 3.29, Cr 5.90, Ni 6.57, As 0.60, Mn 9.30, Zn 147.67, Fe 267.29 µg/g dw) to lowest in *M. penelope* (As 0.13), *C. olor* (Cd 0.78, Cr 2.32, Ni 1.22), *T. totanus* (Fe 115.13), *C. ciconia* (Mn 4.43), *A. anser* (Pb 1.24), and *A. acuta* (Zn 37.40). Element levels varied by feeding guild and avian family: piscivores showed the highest Pb, Cd, Cr, Ni, As and Zn, whereas invertebrate predators had the lowest Cd, As, Zn, Fe. Phalacrocoracidae exhibited the highest overall levels, while Scolopacidae (As, Cd, Fe, Zn), Anatidae (Pb, Ni), and Ciconiidae (Cr, Mn) showed the lowest. Cadmium exceeded 2 µg/g in six species; Cr exceeded 2.8 µg/g in all but *A. anser*, *C. ciconia* and *C. olor*; Pb exceeded 4 µg/g in *P. carbo*, with marginal exceedances in four others. Findings indicate elevated trace metal burdens in wintering waterbirds, suggesting ecological risks associated with trophic exposure and habitat contamination.

## Introduction

Environmental contamination, especially the introduction of trace elements, poses substantial hazards to aquatic ecosystems. These contaminants frequently originate from industrial effluents, agricultural runoff, and municipal waste, ultimately accumulating in aquatic ecosystems and wetlands [1,2]. Although not all trace elements are inherently toxic, they are generally classified as essential (e.g., Fe, Zn) or non-essential (e.g., Cd, Pb). Nevertheless, elevated concentrations of both groups can lead to adverse biological effects [3]. Wetlands, internationally recognized for their ecological importance, provide critical ecosystem services, including water filtration, flood regulation, and habitat for diverse wildlife [4]. These ecosystems are especially vital for both resident and wintering species that rely on them for feeding, breeding, and shelter [5,6]. The presence and health of these birds serve as critical indicators of overall ecosystem integrity. However, their well-being can be compromised by the accumulation of contaminants, including trace elements, which negatively affect habitat quality and the availability of food resources essential for their survival. Nonetheless, elevated concentrations of trace elements within bird populations can result in harmful health impacts, such as reduced reproductive success and increased mortality rates [7,8]. In birds, various tissues are used for health assessment, among which feathers are preferred due to their noninvasive nature, ease of collection, and capacity to provide reliable samples, making feather tissue a key indicator for examining health and trace element concentrations [9,10]. Studies indicate that the chemical composition of feathers is directly linked to dietary intake, environmental conditions, and the overall health status of birds [11–13]. For instance, the concentrations of metals such as Cd and Pb in feather tissue can indicate environmental contamination and serve as effective markers for assessing the general health of avian species [14,15]. Thus, the analysis of feathers has become a valuable tool in ecological research and bird conservation efforts [16,17].

Migratory birds experience diverse environmental conditions throughout their annual cycle, resulting in exposure to varying trace element levels across different habitats [16]. The accumulation of these contaminants can occur in both breeding and wintering habitats, as each presents distinct environmental contaminant profiles and dietary sources [18]. Disentangling the relative contributions of these habitats to the overall tissue burden requires extensive spatial and temporal monitoring. Given the pivotal role of wintering habitats in contaminant exposure [19,20], this study focuses exclusively on migratory birds wintering at the Fereydunkenar International Wetland (FIW) along the southern coast of the Caspian Sea, Iran. This wetland provides habitat for 28 resident species and 63 migratory species.

The behavior and ecological niche of each species play critical roles in their exposure to pollutants. For example, herbivorous birds primarily absorb trace elements from plant material, while carnivorous species acquire these elements through their prey, resulting in different patterns of bioconcentration [10,21]. This variability underscores the importance of understanding species-specific behaviors and trophic levels when assessing pollutant dynamics. The concept of trophic levels is fundamental to understanding how elements move through food webs. Organisms at higher trophic levels generally exhibit higher concentrations of contaminants due to trophic transfer effect, a process in which contaminants become increasingly concentrated as they move up the food chain, especially certain elements such as As, Pb, and Cd, which are well-known for their biomagnification potential. These elements tend to persist and accumulate in organisms, posing significant risks to top predators by affecting their health, reproduction, and survival. [22]. Trophic transfer and biomagnification threaten top predators and serve as key indicators of ecosystem health by revealing contaminant accumulation in the food web [23].

This study was conducted in the winter of 2013 to assess elemental contamination in the Fereydunkenar International Wetland (FIW). Since then, despite the passage of time, a significant lack of published data regarding this critical environmental issue in the southern Caspian Sea region remains. Notably, environmental conditions and land-use patterns around the FIW have remained relatively stable since 2013, with no major industrial developments or substantial land-use changes reported, which supports the continued relevance of the original data [24]. Therefore, the findings of this study provide a valuable foundational framework for assessing elemental contamination in avian populations of this ecologically important habitat.

While previous studies have investigated mercury concentrations in bird feathers in Iran, including the southwestern and northern regions, comprehensive data encompassing multiple trace elements in migratory waterbirds from northern Iran are scarce [25,26]. Given that FIW serves as a crucial wintering ground for Siberian migratory birds in the Middle East, understanding contaminant exposure in these avian populations is of high ecological importance. Consequently, this knowledge gap compelled us to establish our findings as a foundational reference to guide future research and enable accurate long-term monitoring. This will not only assist in tracking changes in contaminant levels but also enhance the understanding of environmental trends affecting bird populations. Ultimately, this work aims to support conservation efforts and inform policy decisions related to habitat protection and contamination control in the region. Relevant studies have been conducted on wintering habitats along the Central Asian Flyway in India and on waterbird species in the Galápagos Islands [20,27].

Therefore, the present study aims to quantify the concentrations of eight trace elements, As, Cd, Cr, Fe, Mn, Ni, Pb, and Zn, in the feathers of eleven migratory bird species wintering in the FIW, located along the southern coast of the Caspian Sea in Iran. We examine species including the Greylag Goose (*A. anser*), Northern Pintail (*A. acuta*), Eurasian Wigeon (*M. penelope*), Mute Swan (*C. olor*), Mallard (*A. platyrhynchos*), Common Pochard (*A. ferina*), Common Redshank (*T. totanus*), White Stork (*C. ciconia*), Common Merganser (*M. merganser*), Great Cormorant (*P. carbo*), and Caspian Tern (*H. caspia*). Furthermore, we evaluate how feeding guild and taxonomic families influence the accumulation patterns of these elements across the studied bird species.

## Materials and methods

### Study site

The FIW, located in northern Iran along the southern coast of the Caspian Sea (36°35’ to 36°45’ N, 52°25’ to 52°35’ E), represents a critical wintering habitat for migratory birds. This region sustains a diverse bird community of 88 species, including 63 migratory species, all of which depend on it to carry out vital activities such as foraging, breeding, and shelter, highlighting its ecological significance. This wetland is composed primarily of harvested rice fields. The unique combination of a flat landscape, high groundwater levels, and seasonal rainfall causes widespread flooding in early autumn, transforming the fields into an ideal habitat for birds. Additionally, several streams flow into the area, further enhancing its ecological value. The fields are bordered by hedges and planted trees, offering a safe and undisturbed refuge for the birds. Beyond the natural resources available in the wetland, local damgah keepers actively contribute to food security for the migratory birds by dispersing substantial quantities of wheat, barley, straw, millet, and other grains. This supplementary feeding practice ensures that the birds have an adequate food supply throughout their stay, supporting the wetland’s role as a vital stopover and wintering site for countless avian species [28].

### Field procedures

This study was conducted in full compliance with the ethical guidelines of the University of Mazandaran and was approved by the Mazandaran Provincial Department of Environmental Protection. The protocol was approved by the Committee on the Ethics of Animal Experiments of the University of Mazandaran (Protocol Number: 33–1724). In February 2013, feather samples were collected from 77 birds belonging to 11 species at the FIW, located in the southern region of the Caspian Sea. Bird species were identified using a field guide for the birds of Iran [29]. Birds were captured using mist nets. A single secondary flight feather was plucked from each wing of every bird to ensure minimal disturbance. These feather samples were carefully placed into labeled plastic bags and coded for identification. The collected samples were then transported to the laboratory for subsequent elemental analysis. Standard protocols for sampling and handling were followed to prevent contamination and ensure the integrity of the samples. Feather samples were stored at 20–25°C with 40–60% relative humidity, protected from light and contaminants in sealed, inert containers to maintain sample integrity before analysis [30].

### Analytical method

In the laboratory, feather samples were carefully processed following established protocols [11,31,32]. First, the feathers were then washed alternately with deionized water and acetone to remove any external contaminants adhering to the surface. The feathers were first washed for 1 hour, then dried in an oven at 80°C. After drying, they were ground into a fine powder, and 0.1 g of the powder was carefully weighed for the digestion process. Digestion was performed in two steps using Teflon tubes. In the first step, the samples were treated with 70% nitric acid and heated at 120°C for 24 hours. In the second step, 1 mL of hydrogen peroxide was added to the samples, which were then left for another 24 hours. The final volume of the digested solution was recorded to calculate the concentration of metals. Blanks were prepared similarly without samples to verify contamination and instrumental precision. For quality control, standard reference material (National Institute of Standards and Technology, NIST 1648e), along with blanks and spiked samples, was run after every 10 samples. The concentrations of As, Cd, Cr, Fe, Mn, Ni, Pb, and Zn in the samples were determined using atomic absorption spectrometry (Thermo Scientific Model 97 GFS). Mean recovery rates for the metals ranged between 95% and 105%, ensuring the reliability of results. The mean recovery rates for all elements in the samples ranged from 93% to 106%, falling within acceptable limits. Additionally, the recovery rates for the certified reference samples were within 10% of their certified values. The detection limits for the analyzed elements were as follows: 0.01 μg g ⁻ ¹ for As, 0.004 μg g ⁻ ¹ for Cd, 0.03 μg g ⁻ ¹ for Cr, 0.05 μg g ⁻ ¹ for Fe, 0.03 μg g ⁻ ¹ for Mn, 0.001 μg g ⁻ ¹ for Pb, and 0.005 μg g ⁻ ¹ for Zn. All concentrations were expressed in μg g ⁻ ¹ on a dry weight basis.

### Data analysis

“Sample sizes (n) differed among species, reflecting variations in species abundance and availability, which in turn affected their representation across feeding guilds and avian families, as shown in the tables.”. Statistical analyses were conducted using SPSS software (version 18). To ensure the validity of parametric tests, the data were assessed for normality using the Shapiro-Wilk test. For the evaluation of differences in feather concentrations of studied trace elements across feeding guilds and avian families, a General Linear Model (GLM) followed by Tukey’s pairwise comparison was employed. A significance threshold of p < 0.05 was used to determine whether element concentrations varied significantly between the different categories. In addition, Pearson correlation analysis was applied to examine the relationships between the concentrations of various elements within the feathers.

## Results

### General trends for elements and species association

Table 1 presents the mean concentrations (± standard deviations) of As, Cd, Cr, Fe, Mn, Ni, Pb, and Zn in the feathers of 11 bird species. Among the analyzed elements, the essential nutrients Zn and Fe consistently exhibited the highest concentrations across all studied species. The mean feather concentrations of Pb ranged from 1.24 µg g ⁻ ¹ in *A. anser* to 5.65 µg g ⁻ ¹ in *P. carbo*. Similarly, Cd levels ranged from 0.78 µg g ⁻ ¹ in *C. olor* to 3.29 µg g ⁻ ¹ in *P. carbo*, while Cr concentrations varied from 2.32 µg g ⁻ ¹ in *C. olor* to 5.90 µg g ⁻ ¹ in *P. carbo*. Nickel concentrations spanned from 1.22 µg g ⁻ ¹ in *C. olor* to 6.57 µg g ⁻ ¹ in *P. carbo*. For As, the lowest concentration was observed in *M. penelope* (0.13 µg g ⁻ ¹), while the highest was found in *P. carbo* (0.60 µg g ⁻ ¹). Manganese levels ranged from 4.43 µg g ⁻ ¹ in *C. ciconia* to 9.30 µg g ⁻ ¹ in *P. carbo*, and Zn concentrations varied widely, from 37.40 µg g ⁻ ¹ in *A. acuta* to 147.67 µg g ⁻ ¹ in *P. carbo*. Iron concentrations were the highest among all measured elements, ranging from 115.13 µg g ⁻ ¹ in *T. totanus* to 267.29 µg g ⁻ ¹ in *P. carbo* (Table 1).

**Table 1:** Trace element concentrations (µg g ⁻ ¹) in bird feathers from the FIW.

Scientific name	n	Feeding guild	As	Cd	Cr	Fe	Mn	Ni	Pb	Zn
*A. anser*	9	Herbivore	0.20 ± 0.05	1.65 ± 0.34	2.68 ± 0.68	152.05 ± 23.00	4.46 ± 0.64	1.55 ± 0.36	1.24 ± 0.33	61.36 ± 10.51
*A. acuta*	10	Herbivore	0.19 ± 0.08	2.73 ± 0.63	4.13 ± 0.73	134.56 ± 30.83	8.01 ± 1.08	2.75 ± 0.51	2.53 ± 0.55	37.40 ± 12.28
*M. penelope*	6	Herbivore	0.13 ± 0.02	1.23 ± 0.14	3.10 ± 0.14	194.78 ± 5.50	6.20 ± 6.05	2.13 ± 0.06	3.78 ± 0.15	66.39 ± 1.74
*C. olor*	6	Herbivore	0.22 ± 0.03	0.78 ± 0.06	2.32 ± 0.12	193.24 ± 13.25	7.43 ± 0.16	1.22 ± 0.08	1.6 ± 0.10	73.14 ± 5.61
*A. platyrhynchos*	6	Omnivore	0.36 ± 0.06	1.72 ± 0.26	3.28 ± 0.83	242.19 ± 27.02	5.34 ± 0.83	4.51 ± 0.51	3.22 ± 0.51	74.32 ± 10.35
*A. ferina*	10	Omnivore	0.48 ± 0.18	2.21 ± 0.59	3.05 ± 0.88	174.28 ± 26.61	7.23 ± 1.29	3.73 ± 0.51	4.13 ± 0.65	94.36 ± 17.45
*T. totanus*	6	Invertebrate predator	0.18 ± 0.02	1.11 ± 0.35	4.26 ± 0.63	115.13 ± 14.51	8.37 ± 1.23	3.26 ± 0.51	2.89 ± 0.55	41.13 ± 4.63
*C. ciconia*	6	Crab and fish predator	0.20 ± 0.07	2.21 ± 0.46	2.73 ± 0.59	219.35 ± 23.65	4.43 ± 0.96	5.04 ± 0.87	4.09 ± 0.84	76.02 ± 12.20
*M. merganser*	6	Piscivorous	0.38 ± 0.07	1.18 ± 0.11	3.03 ± 0.19	170.50 ± 4.88	8.38 ± 0.36	5.00 ± 0.14	2.21 ± 0.14	81.64 ± 4.81
*P. carbo*	6	Piscivorous	0.60 ± 0.17	3.29 ± 0.78	5.90 ± 0.76	267.29 ± 37.02	9.30 ± 2.07	6.57 ± 1.60	5.65 ± 1.12	147.67 ± 15.69
*H. caspia*	6	Piscivorous	0.53 ± 0.12	2.81 ± 0.84	3.87 ± 0.74	163.66 ± 9.75	6.49 ± 1.06	4.34 ± 0.72	4.93 ± 0.93	104.21 ± 11.47

### Taxonomic differences

The analysis revealed significant differences in the concentrations of elements among the examined bird families (p < 0.05) (Table 2). Among the families, Phalacrocoracidae showed the highest levels of all elements. Conversely, the lowest concentrations of Pb and Ni were observed in Anatidae, whereas Scolopacidae exhibited the lowest levels of As, Cd, Fe, and Zn. Similarly, the lowest concentrations of Cr and Mn were recorded in Ciconiidae (p < 0.05). These findings highlight distinct patterns of element concentration across different avian families.

**Table 2:** Trace element concentrations (µg g ⁻ ¹) in bird feathers across different families. Values are presented as mean ± SD, with letters indicating statistically significant differences (p < 0.05).

Family	n	As	Cd	Cr	Fe	Mn	Ni	Pb	Zn
**Anatidae**	53	0.28 ± 0.15^b^	1.77 ± 0.75^c^	3.14 ± 0.82^c^	174.74 ± 38.38^c^	6.73 ± 2.46^b^	2.94 ± 1.34^e^	2.69 ± 1.13^d^	68.73 ± 21.49^c^
**Ciconiidae**	6	0.20 ± 0.07^b^	2.21 ± 0.46^b^	2.73 ± 0.59^d^	219.35 ± 23.65^b^	4.43 ± 0.96^c^	5.04 ± 0.87^b^	4.09 ± 0.84^b^	76.02 ± 12.20^c^
**Phalacrocoracidae**	6	0.60 ± 0.17^a^	3.29 ± 0.78^a^	5.90 ± 0.76^a^	267.29 ± 37.02^a^	9.30 ± 2.07^a^	6.57 ± 1.60^a^	5.65 ± 1.12^a^	147.67 ± 15.69^a^
**Scolopacidae**	6	0.18 ± 0.02^b^	1.11 ± 0.35^d^	4.26 ± 0.63^b^	115.13 ± 14.51^d^	8.37 ± 1.23^a^	3.26 ± 0.51^d^	2.89 ± 0.55^c^	41.13 ± 4.63^d^
**Sternidae**	6	0.53 ± 0.12^a^	2.81 ± 0.84^b^	3.87 ± 0.74^b^	163.66 ± 9.75^c^	6.49 ± 1.06^b^	4.34 ± 0.72^c^	4.93 ± 0.93^a^	104.21 ± 11.47^b^

### Influence of the feeding guild

To investigate the influence of feeding guilds on the concentration of eight trace elements, the avian subjects in this study were categorized into distinct feeding guilds: herbivorous, omnivorous, piscivorous, crab-and-fish predators, and invertebrate predators [29]. The analysis revealed statistically significant differences in elemental concentrations across these various feeding guilds (p < 0.05) (refer to Table 3).

**Table 3:** Influence of feeding guild on trace element concentrations (µg g ⁻ ¹) in bird Feathers. Values are presented as mean ± SD, with letters indicating statistically significant differences (p < 0.05).

Feeding guild	As	Cd	Cr	Fe	Mn	Ni	Pb	Zn
**Crab and fish predator**	0.20 ± 0.07^c^	2.21 ± 0.46^b^	2.73 ± 0.59^c^	219.35 ± 23.65^a^	4.43 ± 0.96^c^	5.04 ± 0.87^b^	4.09 ± 0.84^b^	76.02 ± 12.20^c^
**Herbivore**	0.19 ± 0.06 cd	1.75 ± 0.85^c^	3.16 ± 0.90^b^	162.65 ± 33.92^c^	6.52 ± 2.96^b^	1.99 ± 0.70^e^	2.22 ± 1.00^e^	56.88 ± 16.85^d^
**Invertebrate predator**	0.18 ± 0.02^d^	1.11 ± 0.35^d^	4.26 ± 0.63^a^	115.13 ± 14.51^d^	8.37 ± 1.23^a^	3.26 ± 0.51^d^	2.89 ± 0.55^d^	41.13 ± 4.63^e^
**Omnivore**	0.44 ± 0.16^b^	2.03 ± 0.54^b^	3.14 ± 0.84^b^	199.75 ± 42.68^b^	6.52 ± 1.46^b^	4.02 ± 0.63^c^	3.79 ± 0.74^c^	86.84 ± 17.86^b^
**Piscivorous**	0.50 ± 0.15^a^	2.43 ± 1.12^a^	4.27 ± 1.37^a^	200.48 ± 53.00^b^	8.06 ± 1.75^a^	5.30 ± 1.36^a^	4.26 ± 1.72^a^	111.17 ± 30.22^a^

The mean concentrations of Pb in feathers varied notably among the feeding guilds, with piscivorous birds exhibiting the highest concentration at 4.26 µg g ⁻ ¹, while herbivorous birds displayed the lowest concentration at 2.22 µg g ⁻ ¹. For Cd, piscivorous birds again had the highest mean concentration at 2.43 µg g ⁻ ¹, in contrast to invertebrate predators, which recorded a mean concentration of only 1.11 µg g ⁻ ¹. Chromium concentrations ranged from 4.27 µg g ⁻ ¹ in piscivorous birds to 2.73 µg g ⁻ ¹ in crab-and-fish predators. Nickel concentrations were similarly distributed, with piscivorous birds showing a mean concentration of 5.30 µg g ⁻ ¹, while herbivorous birds had a lower mean concentration of 1.99 µg g ⁻ ¹. Arsenic concentrations were highest in piscivorous birds at 0.50 µg g ⁻ ¹, compared to a lower concentration of 0.18 µg g ⁻ ¹ observed in invertebrate predators. Manganese concentrations varied significantly, with invertebrate predators having the highest mean concentration at 8.37 µg g ⁻ ¹, while crab-and-fish predators had a mean concentration of 4.43 µg g ⁻ ¹. Zinc concentrations also demonstrated a marked difference, with the piscivorous birds showing a mean concentration of 111.17 µg g ⁻ ¹, whereas invertebrate predators had a significantly lower mean concentration of 41.13 µg g ⁻ ¹. Lastly, Fe concentrations ranged from 219.35 µg g ⁻ ¹ in crab-and-fish predators to 115.13 µg g ⁻ ¹ in invertebrate predators.

### Relationship between trace elements

The Pearson correlation analysis of trace elements in bird feathers identified 40 statistically significant relationships among 308 examined pairs (p < 0.05 to p < 0.01) (Table 4). Predominantly, positive correlations emerged among Fe, Mn, and Zn, with Fe consistently displaying strong positive associations with Zn, Mn, As, Ni, and Cd across multiple species. Similarly, Mn correlated positively with Fe, Zn, and As, while Zn frequently showed positive associations with Fe and Mn. In contrast, Cd repeatedly demonstrated negative correlations with Cr, As, and Ni, and Ni was negatively associated with Mn, Zn, and As. Chromium also exhibited negative correlations with Cd and As in multiple cases. Lead showed a mixed pattern of correlations, positively correlating with Fe, Cr, As, and Ni, yet negatively with Mn and Zn. Arsenic frequently correlated positively with Fe, Mn, and Pb while exhibiting negative correlations with Cd, Cr, and Ni.

**Table 4:** Relationships between trace element concentrations in bird feathers.

Species	Correlation	r	Species	Correlation	r
*A. acuta*	Pb – Mn	0.641*	*H. caspia*	Cd – Fe	0.820*
	Cd – Fe	−0.776**		Ni – Fe	0.837*
	Ni – Mn	−0.891**	*C. ciconia*	Pb – Cr	0.903*
	Ni – Zn	−0.651*		Pb – As	0.939**
	As – Fe	0.741*		Cr – As	0.813*
	Mn – Zn	−0.771**		Ni – Mn	0.900*
*A. plathyrhinchos*	Cd – Cr	−0.976**		Mn – Fe	0.934**
	Cd – As	−0.819*		Zn – Fe	0.965**
	Cr – As	−0.819*	*P. carbo*	Pb – Mn	−0.982**
	Ni – As	−0.934**		Pb – Zn	−0.850*
	As – Mn	0.909*		Pb – Fe	0.900*
*A. anser*	As – Mn	0.724*		Cd – As	−0.819*
*M. penelope*	Pb – Fe	0.895*		As – Mn	−0.881*
	Mn – Zn	−0.831*		As – Fe	0.904*
	Zn – Fe	−0.831*		Mn – Fe	−0.950**
*C. olor*	Cr – Mn	0.949**	*M. merganser*	Cr – Mn	0.824
*T. totanus*	Ni – Fe	0.987**		Cr – Zn	0.813
*H. caspia*	Pb – Ni	0.924**		Mn – Zn	0.969**
	Cd – Ni	0.886*		Mn – Fe	0.974**
	Cd – Mn	0.926**		Zn – Fe	0.985**

Pearson’s correlations are reported. **p < 0.01 and *p < 0.05.

## Discussion

### Influence of feeding guild

Dietary habits are widely acknowledged as a key factor influencing trace element bioaccumulation in avian species. Consistent with this, our investigation evaluated the impact of distinct feeding guilds on elemental accumulation by correlating trace element concentrations with the trophic behaviors of the studied bird species, thereby supporting the fundamental role of feeding ecology highlighted in prior avian contaminant studies [31,33–35]. Among the Anseriformes, omnivorous *A. ferina* showed the highest concentration of trace elements compared to other species in this order. This result is consistent with its feeding strategy as a diving duck, with a strong preference for small aquatic invertebrates, including insects, mollusks, crustaceans, worms, amphibians, and small fish [29]. In contrast, species such as *A. anser* and *C. olor*, which are herbivorous [29,36], exhibited significantly lower levels of element concentration as their diet is dominated by plant materials, such as grasses and aquatic vegetation, which are typically less contaminated with trace elements compared to animal-based diets [29,37]. Furthermore, larger-bodied species like *C. olor* may also accumulate fewer elements due to their lower metabolic rates and slower assimilation of contaminants relative to smaller species [38]. Similar findings have been reported in other studies, where smaller birds exhibited higher mercury levels compared to larger bird species due to differences in metabolic activity and exposure pathways [25,26]. Among other Anseriformes, species such as *A. platyrhynchos*, *A. acuta*, and *M. penelope* demonstrated moderate element concentration levels. These species, termed dabbling ducks, feed on both plant and animal materials but show a lower preference for animal prey compared to *A. ferina* [39]. This dietary flexibility likely explains their intermediate levels of element concentration.

When comparing feeding guilds, piscivorous birds, such as members of the Phalacrocoracidae family, exhibited the highest levels of trace element concentration. Within this group, *P. carbo* showed the highest concentration across all elements. This is consistent with the trophic position of piscivorous birds, as they consume larger fish or significant quantities of prey that are higher in the food chain, where contaminants such as trace elements are subject to trophic transfer [40–42]. Trophic transfer has been widely documented in birds that occupy higher trophic levels, as these species are exposed to elevated contaminant levels through their diet [43,44]. For example, Burger [40] reported that piscivorous birds consuming larger fish or higher quantities of contaminated prey tend to have significantly elevated levels of trace elements compared to omnivorous or herbivorous species.

Similarly, invertebrate predators also showed elevated levels of specific elements, particularly Cr and Pb, which are known to accumulate in invertebrates. This finding aligns with previous research suggesting that dietary invertebrate consumption is a key pathway for Cr bioconcentration [40,45]. However, contrary to expectations, Pb levels were not significantly high in invertebrate predators, suggesting varying bioavailability of Pb in invertebrate prey or differences in environmental contamination sources [46,47]. Manganese levels were consistently higher in invertebrate predators than anticipated based on trophic level considerations. This phenomenon may be attributed to the high bioaccumulation capacity of Mn in lower trophic organisms, which are key prey items for these predators [48]. This could be attributed to elevated Mn levels in the invertebrates present in the habitat of these species, although further studies are required to verify this hypothesis. Similar patterns of dietary influence on element concentration have been reported by previous studies, reinforcing the idea that feeding behavior is a major factor affecting contaminant exposure in birds [10,21].

### Taxonomic affiliation

Among examined families, generally the highest and lowest concentration of elements were found in Phalacrocoracidae and Scolopacidae, respectively. It is worth mentioning that at first glance, it might be expected that the members of the Anatidae family would have the lowest concentration of elements due to their herbivorous habits, but the results showed otherwise. When examining the feeding strategies of this family, it becomes evident that some species, such as *A. ferina* and *A. platyrhynchos*, are omnivorous, which contributes to an increased average concentration of elements within the family. Additionally, during the breeding season, these species shift their diet towards insects and aquatic invertebrates to satisfy the heightened energy requirements associated with reproduction [19,29]. This change of strategy can also be a reason for the relatively high element concentration of in members of this family. Another possible explanation lies in the ecological niche occupied by different species within the same habitat. For example, Phalacrocoracidae, as piscivorous birds, occupy a higher trophic level and are more exposed to biomagnification of certain trace elements through the food chain [41]. Similarly, species that forage in benthic environments, where trace elements tend to accumulate in sediments, may experience increased exposure compared to those that primarily feed in open water or on terrestrial resources [49]. Furthermore, interspecific differences in metabolism rates, foraging duration, prey selection, and exposure to contaminated microhabitats within the same area could also contribute to the observed variation in metal concentration [1,50]. Such factors underscore the importance of both trophic positioning and behavioral ecology in determining trace elements bioconcentration among avian species.

Generally, looking at the feeding behavior of the studied families, it is clear that the most important factor in the concentration of elements in their feathers can be feeding strategy. Thus, predatory and piscivores Phalacrocoracidae had the highest concentrations. Other factors like metabolism, mobility, and migratory behavior can also significantly affect the bioaccumulation of elements [51].

Taxonomic differences in trace element accumulation among bird families have been rarely studied, with most research focusing primarily on mercury. Abbasi et al. [34] analyzed eight metals in feathers from the Anatidae, Motacillidae, and Sturnidae families, finding distinct metal profiles linked to their habitat and feeding behaviors: Anatidae had the highest levels of Pb, Cr, and Cu; Motacillidae had the highest Cd and Fe; and Sturnidae exhibited the greatest Ni, Mn, and Zn concentrations. Studies by Mashroofeh et al. [25] and Zolfaghari et al. [26] in Iran showed that mercury concentrations were highest in Falconidae, Accipiteridae, and Strigidae (carnivorous and predatory families) and lowest in the herbivorous Phasianidae, highlighting trophic position and diet as key factors in mercury bioaccumulation.

### Toxic elements and adverse effects

The As concentrations observed in this study, ranging from 0.13 µg g ⁻ ¹ in *M. penelope* to 0.6 µg g ⁻ ¹ in *P. carbo*, were considerably lower than the threshold levels (1.3 µg g ⁻ ¹) at which biological effects are typically anticipated [8]. There are only limited data on As concentrations in waterbirds’ feathers. Additionally, these values were below the average concentration of 21.4 µg g ⁻ ¹ reported by Abdullah et al. [52] for avian feathers in Pakistan, 4.78 µg g ⁻ ¹ by He et al. [53] for a region in Southern China. In contrast, our findings were higher than those recorded in *Larus crassirostris* (0.3 µg g ⁻ ¹) collected from South Korea [54].

Burger and Gochfeld [55] considered Cd concentration of 2 µg g^-1^ as a threshold concentration in feathers that may have an adverse effect on kidneys. The mean Cd concentrations measured in the feathers of *A. acuta, A. ferina, C. ciconia, M. merganser, P. carbo, H. caspia* in the current study exceeded the threshold for adverse effects. For other species, mean concentrations were below the threshold. These findings are consistent with other studies that reported high accumulation of Cd in birds’ feathers [52,56]. However, the Cd concentrations in our study exceeded those reported for four waterbird species from the Galapagos Islands [27], although they were lower than those found in *Fulica atra* and *A. strepera* from two wetlands in Pakistan [57].

Chromium concentration of 2.8 µg g^-1^ in bird feathers may be associated with adverse effects [55]. Except for *A. anser*, *C. ciconia* and *C. olor*, Cr levels for other species were above the threshold level for effects. Mean concentration of Cr measured in the current study was higher than those reported for *Calidris canutus*, *C. pusilla* and *C. alba* from Delaware Bay, USA [58] as well as *Passer montanus* from Anhui Province, China.

Lead concentrations of 4 µg g^-1^ in feathers were found to be associated with delayed parental and sibling recognition, impaired thermoregulation, locomotion, depth perception, feeding behavior, and lowered chick survival in gulls [31]. Mean Pb concentration reported in the current study was well above that threshold level in *p. carbo* and slightly above in *A. ferina*, *C. ciconia*, *M. merganser* and *H. caspia.* On the other hand, Mendes et al. [59] and Burger and Gochfeld [60] suggested the range of 0.51–1.68 µg g^-1^ (dry mass) as a normal background concentration of Pb in feathers of seabirds. Except for *A. anser* and *C. olor* in the current study, other species exceeded this range. Consequently, the levels of Pb observed in the studied birds could pose a potential concern. Moreover, the average concentrations in the other species were considerably lower than thresholds associated with harmful effects. The mean Pb concentration in the current study ranged from 1.24 µg g^-1^ for *A. anser* to 5.65 µg g^-1^ for *P. carbo* and was higher than those reported in the study of Burger and Gochfeld, [55] in feathers of twelve species of seabirds from Midway Atoll in the northern Pacific Ocean and below the mean concentration of 9.03 µg g^-1^, reported from feathers of 50 species of shorebirds of Central Asian Flyway wintering grounds [20].

### Relationship between trace elements

Pearson correlation analysis indicated that out of 308 elemental pairs examined in bird feathers, 40 pairs showed statistically significant relationships (p < 0.05 to p < 0.01). These findings demonstrate complex inter-element interactions, despite the predominantly independent accumulation of most elements. Abbasi et al. [34] further reported that this occurrence may be attributed to the relatively low threshold concentrations of many elements, insufficient to trigger the organism’s regulatory and protective mechanisms. Strong positive correlations were observed between Fe, Mn, and Zn, likely reflecting shared accumulation pathways [61]. This pattern aligns with the established biological roles of these elements in avian metabolism, where they play essential roles in enzymatic activity and critical biochemical processes [62]. For example, elements such as Zn and Cu are known to play key roles in biological systems and may co-accumulate due to their involvement in similar metabolic processes [63]. Durkalec et al. [64] reported similar co-accumulation patterns and significant elemental correlations in birds. The significant correlations observed likely reflect shared sources or similar pathways of incorporation into the feathers, which could be influenced by environmental exposure, diet, or physiological processes during feather formation [13]. Cadmium in bird feathers exhibited significant negative correlations with Cr, As, and Ni. These negative correlations are consistent with established mechanisms of elemental competition for binding sites and activation of detoxification pathways in organisms [65]. Nickel also showed negative correlations with Mn and Zn, likely reflecting competitive interactions for limited absorption sites or overlapping metabolic pathways [66]. Consequently, these negative relationships suggest antagonistic regulation of metal uptake and accumulation in tissues. Similar negative correlations between trace elements have also been reported by other researchers [67,15].

## Conclusion

This study demonstrates that migratory waterbirds wintering in the southern Caspian Sea are exposed to varying trace element levels, with trophic position as a key factor in bioaccumulation. Elevated concentrations of As, Cd, and Pb in piscivorous species illustrate biomagnification and the vulnerability of higher trophic levels to toxicants. Detection of Cd, Cr, and Pb at potentially harmful exposure levels in several species raises concerns about sub-lethal physiological effects. These results highlight the need for ongoing ecotoxicological monitoring in wetlands such as the FIW, a crucial habitat along the Central Asian Flyway. Serving as a foundation for future assessments, this study underscores the importance of avian biomonitoring in regional conservation and pollution control frameworks. Given the Caspian region’s ecological significance and stable land use, targeted mitigation and stronger transboundary cooperation are essential to reduce contaminant risks and protect avian biodiversity. Future research should prioritize long-term trace element monitoring, seasonal and migratory pattern analysis, habitat-specific bioavailability studies, and investigations into the combined effects of multiple trace elements on bird health, especially at higher trophic levels.
